# Associations between access to healthcare, environmental quality, and end-stage renal disease survival time: Proportional-hazards models of over 1,000,000 people over 14 years

**DOI:** 10.1371/journal.pone.0214094

**Published:** 2019-03-21

**Authors:** Marissa B. Kosnik, David M. Reif, Danelle T. Lobdell, Thomas Astell-Burt, Xiaoqi Feng, John D. Hader, Jane A. Hoppin

**Affiliations:** 1 Toxicology Program, Department of Biological Sciences, North Carolina State University, Raleigh, North Carolina, United States of America; 2 Bioinformatics Research Center, North Carolina State University, Raleigh, North Carolina, United States of America; 3 Center for Human Health and the Environment, North Carolina State University, Raleigh, North Carolina, United States of America; 4 National Health and Environmental Effects Research Lab, U.S. EPA, Chapel Hill, North Carolina, United States of America; 5 Population Wellbeing and Environment Research Lab, School of Health and Society, Faculty of Social Sciences, University of Wollongong, Wollongong, New South Wales, Australia; 6 Menzies Centre for Health Policy, University of Sydney, Sydney, New South Wales, Australia; 7 School of Public Health, Peking Union Medical College and The Chinese Academy of Medical Sciences, Beijing, China; 8 ICF, Fairfax, Virginia, United States of America; University of Mississippi Medical Center, UNITED STATES

## Abstract

Prevalence of end-stage renal disease (ESRD) in the US increased by 74% from 2000 to 2013. To investigate the role of the broader environment on ESRD survival time, we evaluated average distance to the nearest hospital by county (as a surrogate for access to healthcare) and the Environmental Quality Index (EQI), an aggregate measure of ambient environmental quality composed of five domains (air, water, land, built, and sociodemographic), at the county level across the US. Associations between average hospital distance, EQI, and survival time for 1,092,281 people diagnosed with ESRD between 2000 and 2013 (age 18+, without changes in county residence) from the US Renal Data System were evaluated using proportional-hazards models adjusting for gender, race, age at first ESRD service date, BMI, alcohol and tobacco use, and rurality. The models compared the average distance to the nearest hospital (<10, 10–20, >20 miles) and overall EQI percentiles [0–5), [5–20), [20–40), [40–60), [60–80), [80–95), and [95–100], where lower percentiles are interpreted as better EQI. In the full, non-stratified model with both distance and EQI, there was increased survival for patients over 20 miles from a hospital compared to those under 10 miles from a hospital (hazard ratio = 1.14, 95% confidence interval = 1.12–1.15) and no consistent direction of association across EQI strata. In the full model stratified by average hospital distance, under 10 miles from a hospital had increased survival in the worst EQI strata (median survival 3.0 vs. 3.5 years for best vs. worst EQI, respectively), however for people over 20 miles from a hospital, median survival was higher in the best (4.2 years) vs worst (3.4 years) EQI. This association held across different rural/urban categories and age groups. These results demonstrate the importance of considering multiple factors when studying ESRD survival and future efforts should consider additional components of the broader environment.

## Introduction

The prevalence of end-stage renal disease (ESRD) in the United States (US) increased from 1,095 to 1,748 prevalent cases per million between the years of 2000 and 2013 with a five-year survival of 42% for patients on hemodialysis [1]. In the US, ESRD disproportionately affects racial and ethnic minorities with socioeconomic and environmental factors suspected to play a role [2–5]. Similar disparities can also be found with patient access to and quality of care with numerous studies reporting inequalities in nephrology services for socially disadvantaged adults [2,3,5–8]. Further, impacts of geographic variability in access to care for kidney disease survival are well documented. Greater risks for mortality have been observed with increasing distance between patient residences and nephrologists [9–12] with the added variability of quality of nephrology services offered further influencing survival [13,14]. While these studies have assessed influences on patient survival time relative to distance from kidney care, this does not address the influence of access to care for earlier health issues that may progress to ESRD. Early detection of ESRD is expected to delay adverse outcomes [15–17] so assessing patient survival based on distances to facilities utilized after diagnosis may not capture the full influence of patient access to care on ESRD survival time.

Multiple studies have shown hospital utilization to be related to patient distance [18–20], but associations between access to care and survival are varied depending on the outcome under study [21–24]. In a review of 108 studies analyzing the association between travel time/distance to health care and adverse patient outcomes, Kelly et al. found evidence of a positive association in 77% of articles with others showing no association or an inverse association [23]. In studies evaluating the role of hospital distance on survival, the focus tends to be on the need for rapid emergency services [21,22,24] or on distance traveled following the diagnosis of a disease [25–27] rather than the impact that distance could have for all health care needs leading to disease development. By studying more general health facilities such as hospitals for patients who have lived in one geographic region their whole life, the role that geographic access to care (rather than utilization, *per se*) plays in the pre- and post-diagnosis progression and development of ESRD may be better captured. Further, because poorer socioeconomic status is a risk factor for ESRD [2,28,29] and studies have shown increased hospital utilization among these groups relative to those of higher socioeconomic status [18,30–32], hospitals may be a good measure for access to care when analyzing associations with survival time for patients with ESRD.

Geographic variability has been observed for both the incidence and prevalence of ESRD with population-adjusted incidence rates from 2014 being highest in the Ohio Valley, Texas, California, and the Southeast [1,2]. This geographic variability suggests some environmental exposures may be determinants of ESRD, but the research on the impact of these factors is limited [2,33,34]. For instance, there is some evidence to support an association between pesticide exposure and ESRD development [33,35], but concomitant exposures that could be contributing to ESRD have been understudied [36]. The sociodemographic environment is also suspected to contribute to ESRD development, particularly due to the higher rates among racial minorities and the socially disadvantaged [2,5,8]. Despite the apparent contributions from multiple environmental components influencing ESRD etiology, studies elucidating the role that these aggregate exposures have on survival are limited.

The influence of environmental factors on any disease morbidity and mortality has been shown to vary based on features of the physical environment. Still, studies are limited on the effects of these multiple exposures in tandem and their correspondent health implications [37–39]. The Environmental Quality Index (EQI) is an aggregate measure of ambient environmental quality and was constructed at the county-level across the US. It is composed of five domains (air, water, land, built, and sociodemographic), each constructed to represent exposures within that domain [40,41]. The EQI provides a means to relate the overall environment to human health and has been used to investigate associations between environmental quality and mortality rates in the US [39], cancer incidence rates [42], and infant mortality rates [43]. As a measure of environmental influence on disease progression and survival encompassing different components of the environment, the EQI could offer insight into ESRD survival.

Factors contributing to patient survival from a disease are often encountered before the disease diagnosis, yet understanding of the role that the overall environment plays in this survival is limited. Here we analyze patient data in the U.S. Renal Data System (USRDS) to evaluate associations between survival in people living with ESRD following diagnosis and the broader environment by analyzing access to care and the EQI. By analyzing the role that these two elements play in ESRD survival, we elucidate the potential impacts that the overall environment has on ESRD survival and demonstrate the importance of analyzing multiple components of the environment when studying complex diseases.

## Materials and methods

### Patients

The USRDS is the largest and most comprehensive national ESRD surveillance system in the US [44]. The USRDS contains data on all ESRD cases in the US through the Medical Evidence Report CMS-2728 which is mandated for all new patients diagnosed with ESRD [45]. Detailed information about the USRDS can be found on their website (http://www.usrds.org).

We used the 2016 USRDS core patient, medical evidence, and residency Standard Analysis Files for analysis in this study [46]. Data can be requested from USRDS. Written consent was obtained from North Carolina State University Institutional Review Board for the use of human subjects’ data. We identified 1,535,798 cases of ESRD diagnosed between January 2000 and December 2013 using the USRDS-derived first ESRD service date. Patients whose death date was the same as their first ESRD service date were excluded (N = 6,064; 0.4%). We excluded patients who were under 18 at their first ESRD service date (N = 14,034; 0.9%) as well as those whose USRDS patient, medical evidence, and residency files were missing key study covariates: race, gender, age, body mass index (BMI), current alcohol dependence, and current tobacco use, or residency information (N = 46,990, 3.0%). This left us with 1,468,710 patients for whom to determine average distance to the nearest hospital. Patients who had any residency outside of the US were excluded (N = 26,442, 1.8%). Patients who did not have a zip code or county matching any on record were excluded (N = 5,334; 0. 4%), along with those who had a zip code that did not correspond to the same state as the county code (N = 2,189, 0. 2%). Because both the EQI and the hospital distance determinations were at the county level, any patients with residency files from multiple counties were excluded from the study (N = 342,464, 23.9%). The demographic makeup of the patients who moved between counties was similar to those that remained in one county for the duration of their lives, however median survival was greater by 3 months for patients who moved compared to those who did not move. Our final dataset included 1,092,281 patients.

### Average county hospital distance determinations

Multiple studies have shown straight-line (Euclidean) distance and actual travel distance over a road network to be highly correlated [47–53]. Additionally, because our study covers a 14 year timeframe, the road networks for any given year would vary. The U.S. Department of Transportation Federal Highway Administration estimates an additional 6,500 miles of roads were built each year between 1980 and 2008 with additional increases in lanes of traffic and public road bridges [54]. For these reasons, we used average distance to a hospital as a measure of access to care. The average distance to the nearest hospital within each county in the US was determined using the straight-line projected distance using geographic information systems (GIS) software package suite ArcGIS version 10.5.1 [55]. A list of addresses for all hospitals registered with Medicare in the US (last updated January 2018) was downloaded from the Medicare website [56]. 93% of these addresses included latitudes and longitudes. We used Google Earth to determine the geographic locations of the hospitals without these coordinates, and converted these locations to latitude-longitude coordinates within the GIS software. Using this methodology, all but one of the 4,747 hospitals were used in our analysis. A 0.05 degree latitude-longitude grid of points was overlaid across the US, and the distance to the nearest hospital was determined for each of these roughly 1.2 million grid points. Using 2010 U.S. Census county lines [57], we determined the county in which each of these grid points was located. This analysis was performed separately for Alaska, Hawaii, and the continental U.S., employing 0.05 degree grids and Albers equal area conic projections specific to each of these three locations.

The average distance to the nearest hospital within each county was taken as the mean of the distances to the closest hospital for all of the grid points that fell within the county. This methodology enabled the determination of the distance to the nearest hospital from many places within a county, disregarding whether the hospital was in the same county or state as the county in question. We conducted this analysis at the county level to match the geographic level of analysis of the EQI. The county FIPS codes and corresponding average distance to the nearest hospital from the GIS analysis were matched to the county FIPS codes corresponding to the USRDS patient data. If a patient’s county FIPS code did not match a county FIPS code from the GIS analysis, then the patient’s zip code was used to identify the county FIPS code in the HUD-USPS Crosswalk Files [58]. In total, there were 3,111 counties in our study. Average distance to the nearest hospital for patients was stratified into three groups: those living in a county with an average distance of under 10 miles, between 10–20 miles, and over 20 miles to the nearest hospital. These intervals were selected to cover patients with a hospital nearby (under 10 miles away) and patients in a more remote location (hospital over 20 miles away). These bins were in accordance with definitions of remote locations and percentiles used in other studies developing intervals for distance to care [9,10,59].

### Environmental Quality Index

The EQI was used as a measure of cumulative environmental quality for counties across the US. The development of the EQI has been well described [40,41]. In brief, the EQI was constructed for 2000–2005 for all US counties and is composed of five domains (air, water, built, land, and sociodemographic), each composed of variables to represent the environmental quality of that domain. Domain-specific EQIs were developed using principal components analysis (PCA) to reduce these variables within each domain while the overall, total EQI was constructed from a second PCA from these individual domains [41]. To account for differences in environment across rural and urban counties, the overall and domain-specific EQIs were stratified by rural urban continuum codes (RUCCs) [60]. RUCCs are assigned by the USDA and describe how metropolitan or rural an area is using nine-item categories and these RUCCs were binned into four categories with RUCC1 being the most metropolitan urbanized and RUCC4 being the most rural [41]. These RUCC-stratified EQIs were in addition to the overall EQI. These EQI data were downloaded from the U.S. EPA [61]. Higher EQI values correspond to “worse” environmental quality, in that the variables used to construct the EQI were associated with adverse health outcomes or ecologic effects [41].

All patients were assigned an EQI using county FIPS codes. EQI values for the total, overall EQI domain as well as the five individual domains were grouped into quantiles according to the distribution of the overall EQI: 0-5^th^ percentile, 5^th^-20^th^ percentile, 20^th^-40^th^ percentile, 40^th^-60^th^ percentile, 60^th^-80^th^ percentile, 80^th^-95^th^ percentile, and 95^th^-100^th^ percentile, with lower percentiles corresponding to better EQI values. These quantiles were selected to capture the extremes of the “best” and “worst” EQIs and demonstrate the wide variability in EQIs across the US [41]. In addition to the overall EQI and the five domains, the four RUCC-stratified overall EQIs were grouped into the same percentiles according to their individual distributions.

### Statistical analysis

Patients were followed from their first ESRD service date (diagnosis) until death, date of first transplant, or the end of the study time (31 December 2013). The total period of observation was 14.0 years. Cox proportional hazards models were used to calculate hazard ratios (HRs) for risk of mortality from ESRD for hospital distance and EQI after adjustment for covariates; years of survival following diagnosis was the timescale. Covariates included were: gender, race (African American, Asian, Caucasian, Native American, or Other, as reported in the USRDS patient file), mean BMI from follow-up visits after diagnosis (underweight (below 18.5), normal weight (between 18.5 and 24.9), overweight (between 25 and 29.9), or obese (30 or over)), alcohol use after ESRD diagnosis (as reported in the USRDS medical evidence report), tobacco use after ESRD diagnosis (as reported in the USRDS medical evidence report), county-level RUCCs to account for differences in rurality (RUCC1-RUCC4, most urban to most rural), and age at first ESRD service date (between 18 and 39, between 40 and 65, over 65 years). Separate models were generated with additional adjustment for either average distance to the nearest hospital (under 10 miles, between 10 and 20 miles, or over 20 miles) or the overall, total EQI (0-5^th^ percentile, 5^th^-20^th^ percentile, 20^th^-40^th^ percentile, 40^th^-60^th^ percentile, 60^th^-80^th^ percentile, 80^th^-95^th^ percentile, or 95^th^-100^th^ percentile). The full model included all of the covariates with average distance to the nearest hospital and the total EQI mutually adjusted. This full model was then stratified by average distance to the nearest hospital, and the resultant model was further stratified separately by age and rurality with EQI quantiles developed using the RUCC-stratified total EQIs. Additional models were prepared for each of the five EQI domains (air, water, land, built, and sociodemographic) individually, with mutual adjustment for average distance to the nearest hospital, and with the model stratified by average distance to the nearest hospital.

Adjusted survival curves were generated using the corrected group prognosis method which generates survival curves for each combination of covariates in the data using the proportional hazards coefficients and takes a weighted average of these curves proportional to the number of individuals for each combination [62]. Median and 90^th^ percentile survival times were determined from these adjusted survival curves with significance determinations based on the proportional hazards coefficients used to develop the curves; 90^th^ percentiles are defined as 90% of patients have reached time of death. Survival analysis was completed using the R/survival package [63]. All analyses were done using R version 3.3.2 [64].

## Results

### Patient characteristics

Patient demographics organized by distance to the nearest hospital are summarized in Table 1. Of the 1,092,281 patients, 783,857 (72%) lived under 10 miles from a hospital while 55,460 (5.1%) lived over 20 miles from a hospital. Each hospital distance group was similar for all demographics except Native American race and rurality. For Native Americans, 37% lived over 20 miles from the nearest hospital compared to 1.5–6.2% for non-Native American races. For patients living farther from a hospital, the majority live in more rural communities compared to those who live closer to a hospital. The majority of patients with hospitals in the most rural regions are between 10–20 miles away (59.3%). The best EQI percentiles correspond more with rural regions while the worst EQIs overlap more with urban regions. Over 600,000 patients (>50%) lived in the two strata corresponding to worst environmental quality (80-95^th^ percentile, 95^th^-100^th^ percentile) with 100,000 patients (~10%) living in the three best EQI strata (0-5^th^ percentile, 5^th^-20^th^ percentile, 20^th^-40^th^ percentile).

**Table 1:** Demographic characteristics of 1,092,281 ESRD patients from 2000 to 2013 stratified by distance to the nearest hospital.

		USRDS patients (n = 1,092,281)		
	% total patients in each demographic	% patients in each hospital group		
		Under 10mi	10-20mi	Over 20mi
Gender				
Male	55.8	55.8	55.6	57.1
Female	44.2	44.2	44.4	42.9
Race				
Caucasian	65.8	62.6	72.5	79.9
African American	28.6	31.9	22.6	8.4
Asian	4.2	4.7	2.9	3.5
Native American	1.1	0.4	1.8	7.8
Other	0.4	0.4	0.2	0.3
Age at first ESRD service date				
18–39	7.9	7.9	7.8	8.4
40–65	44.4	44.0	45.0	48.2
Over 65	47.7	48.1	47.2	43.4
BMI				
Normal weight	32.3	32.6	31.3	32.1
Underweight	3.9	4.1	3.6	3.4
Overweight	28.5	28.3	28.9	29.9
Obese	35.3	35.1	36.1	34.6
Current alcohol dependence				
No	98.4	98.4	98.4	98.3
Yes	1.6	1.6	1.6	1.7
Current tobacco use				
No	94.1	94.2	93.8	94.7
Yes	5.9	5.8	6.2	5.3
Rurality				
Most Urban	83.1	88.2	69.1	76.2
Urban	7.1	5.4	12.1	9.0
Rural	8.1	5.8	14.7	11.2
Most Rural	1.6	0.7	4.2	3.7

All patients lived in one county for the duration of their lifetime.

^a^Hospital group defined as average distance to the nearest hospital.

### Total population-based analyses

We developed two separate proportional-hazards models to elucidate the individual effects of hospital distance and EQI on survival time. Covariates included in both models were gender, race, BMI, alcohol use, tobacco use, rurality, and age. When we added average distance to the nearest hospital into our model (Table 2), we found increased survival time for patients living over 20 miles from the nearest hospital compared to under 10 miles (hazard ratio (HR) = 0.90, 95% confidence interval (CI) = 0.89–0.91). Separately, when we added EQI into our model (Table 2), we found no significant difference in survival in the worst EQI compared to the best EQI (HR = 1.01, 95% CI = 0.98–1.05). Stratifying these separate models into rural versus urban categories did not change these associations (not shown).

**Table 2:** Proportional hazard model results for ESRD survival by hospital distance and EQI (1,092,281 patients, 2000–2013).

		Hospital Distance	EQI
		HR (95% CI)^a^	HR (95% CI)
Hospital Distance	Under 10 miles	Ref	-
	10–20 miles	0.98 (0.97–0.98)	-
	Over 20 miles	0.90 (0.89–0.91)	-
EQI Category	EQI 0–5% (best)	-	Ref
	EQI 5–20%	-	1.09 (1.05–1.13)
	EQI 20–40%	-	1.11 (1.07–1.15)
	EQI 40–60%	-	1.11 (1.07–1.15)
	EQI 60–80%	-	1.11 (1.07–1.15)
	EQI 80–95%	-	1.08 (1.04–1.12)
	EQI 95–100% (worst)	-	1.01 (0.98–1.05)
Race	Caucasian	Ref	Ref
	African American	0.80 (0.80–0.81)	0.80 (0.80–0.81)
	Asian	0.59 (0.58–0.59)	0.60 (0.59–0.61)
	Native American	0.82 (0.80–0.84)	0.79 (0.77–0.81)
	Other	1.24 (1.20–1.29)	1.25 (1.20–1.30)
Gender	Male	Ref	Ref
	Female	1.01 (1.01–1.02)	1.01 (1.01–1.02)
BMI	Normal weight	Ref	Ref
	Underweight	1.31 (1.29–1.32)	1.31 (1.29–1.32)
	Overweight	0.85 (0.85–0.86)	0.85 (0.85–0.86)
	Obese	0.82 (0.81–0.82)	0.82 (0.81–0.82)
Alcohol Dependence	No	Ref	Ref
	Yes	1.23 (1.20–1.25)	1.23 (1.21–1.25)
Tobacco Use	No	Ref	Ref
	Yes	1.11 (1.10–1.12)	1.11 (1.10–1.12)
Rurality	Most Urban	Ref	Ref
	Urban	1.08 (1.07–1.09)	1.04 (1.03–1.06)
	Rural	1.09 (1.08–1.10)	1.04 (1.03–1.06)
	Most Rural	1.14 (1.12–1.16)	1.10 (1.08–1.12)
Age at first ESRD service date	18–39	Ref	Ref
	40–65	2.05 (2.02–2.08)	2.05 (2.02–2.08)
	Over 65	4.26 (4.20–4.32)	4.27 (4.21–4.33)

^a^HR (95% CI) = Hazard ratio (95% Confidence Interval).

**Fig 1:**
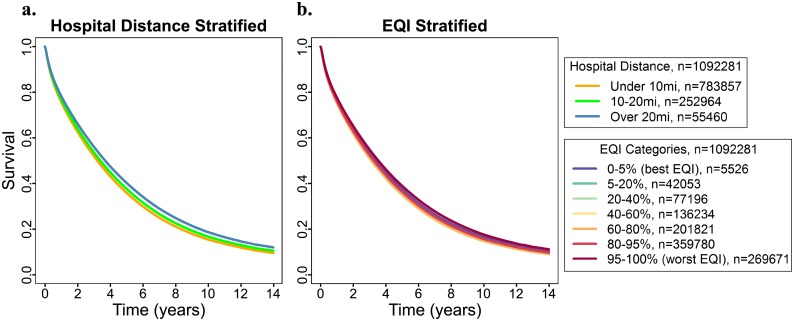
Adjusted survival curves for all patients developed using the full model mutually adjusted for average distance to the nearest hospital and total EQI. (a) Quantiles were generated using distance from the nearest hospital, (b) Quantiles were generated using total EQI.

Our full model included distance from the nearest hospital and EQI mutually adjusted to assess possible effects on survival time in patients diagnosed with ESRD. Two adjusted survival curves were generated from the full model: one stratified by distance to the nearest hospital (Fig 1a) and one stratified by total EQI (Fig 1b). Adding total EQI into the model did not affect the inverse association between survival and distance to the nearest hospital (Over 20 miles compared to under 10 miles HR = 0.88, 95% CI = 0.87–0.89). Adding hospital distance into the model did not influence the associations between environmental quality and patient survival time as there was no substantial difference between the best and worst EQI strata (Fig 1b). Repeating this analysis with the five individual domains of the EQI instead of the overall EQI did not clarify these associations. Just as with the total EQI, survival time was significantly greater farther from the nearest hospital. The direction of association between the EQI and individual domains was inconsistent with no significant difference between the best and worst EQI percentiles for the air and built EQI domain and an improvement in survival in worse EQI percentiles compared to the best EQI percentile for the water and land EQI domain. While survival was decreased in the worst EQI percentiles compared to the best EQI percentile for the sociodemographic domain, there was no uniformity to this association between the remaining strata (data not shown).

### Hospital-distance stratified analyses

**Fig 2:**
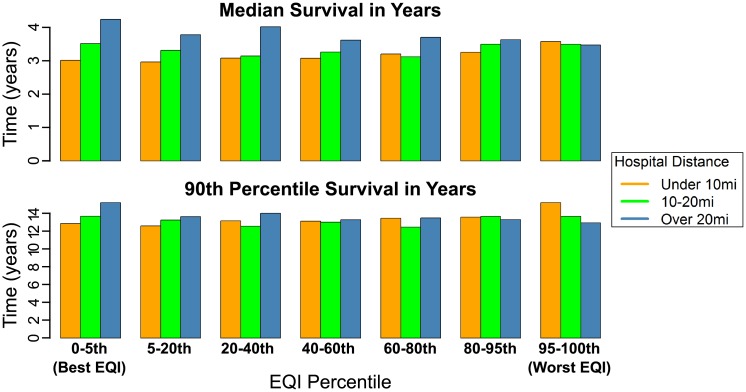
Bar graphs for patient median and 90^th^ percentile survival for different total EQI domains from the full model separated by average distance to the nearest hospital. Quantile 1 = best EQI, Quantile 7 = worst EQI. Orange = Patients with nearest hospital under 10 miles away, Green = Patients with nearest hospital 10–20 miles away, Blue = Patients with nearest hospital over 20 miles away. Hazard ratios (Table 3) were used with the corrected group prognosis method to generate adjusted survival curves and generate medians/90^th^ percentile survival.

To further analyze the role of the broader environment on ESRD survival, we ran the full model with the total EQI stratified by hospital distance (Fig 2, Table 3- all medians/90^th^ percentiles determined from corrected group prognosis method adjusted survival curves generated using these proportional hazard model coefficients). We saw a negative association between patient survival and better EQIs for patients living under 10 miles from the nearest hospital, and a positive association between patient survival and better EQIs for patients living over 20 miles from the nearest hospital. For patients under 10 miles from the nearest hospital, median survival was 2.97 years (90^th^ percentile 12.69 years) in the best EQI compared to a median of 3.53 years (90^th^ percentile over 14 years, survival beyond 2000–2013 study time) in the worst EQI. This association is flipped for patients living over 20 miles from the nearest hospital: median survival was 4.18 years in the best EQI (90^th^ percentile over 14 years, survival beyond 2000–2013 study time), and 3.43 years in the worst EQI (90^th^ percentile 12.77 years). For patients living between 10 and 20 miles from the nearest hospital, we saw no uniform direction of association between better EQIs and patient survival: median survival was 3.47 years in the best EQI (90^th^ percentile 13.50 years) and 3.45 years in the worst EQI (90^th^ percentile 13.48 years).

**Table 3:** Hazard ratios for ESRD and total EQI stratified by distance to the nearest hospital (1,092,281 patients, 2000–2013).

		Hospital Distance		
		Under 10 miles (N = 783,857)	10–20 miles (N = 252,964)	Over 20 miles (N = 55,460)
		HR (95% CI)^a^	HR (95% CI)	HR (95% CI)
EQI Category	EQI 0–5% (best)	Ref	Ref	Ref
	EQI 5–20%	1.02 (0.92–1.12)	1.06 (1.01–1.11)	1.12 (1.03–1.23)
	EQI 20–40%	0.98 (0.89–1.08)	1.11 (1.06–1.16)	1.06 (0.97–1.14)
	EQI 40–60%	0.98 (0.89–1.09)	1.07 (1.03–1.12)	1.17 (1.08–1.27)
	EQI 60–80%	0.95 (0.85–1.05)	1.12 (1.07–1.17)	1.15 (1.05–1.25)
	EQI 80–95%	0.93 (0.84–1.03)	1.00 (0.96–1.05)	1.17 (1.07–1.27)
	EQI 95–100% (worst)	0.85 (0.77–0.94)	1.00 (0.96–1.06)	1.22 (1.10–1.35)
Race	Caucasian	Ref	Ref	Ref
	African American	0.78 (0.78–0.79)	0.82 (0.81–0.83)	0.86 (0.82–0.90)
	Asian	0.58 (0.57–0.59)	0.63 (0.61–0.66)	0.66 (0.61–0.70)
	Native American	0.92 (0.88–0.97)	0.83 (0.79–0.86)	0.74 (0.71–0.77)
	Other	1.20 (1.16–1.25)	1.32 (1.19–1.45)	1.70 (1.44–2.01)
Gender	Male	Ref	Ref	Ref
	Female	1.02 (1.01–1.02)	1.00 (0.99–1.02)	0.99 (0.97–1.02)
BMI	Normal weight	Ref	Ref	Ref
	Underweight	1.30 (1.28–1.32)	1.32 (1.28–1.35)	1.33 (1.25–1.41)
	Overweight	0.85 (0.84–0.86)	0.85 (0.84–0.86)	0.87 (0.84–0.89)
	Obese	0.82 (0.81–0.82)	0.81 (0.80–0.82)	0.83 (0.81–0.85)
Alcohol Dependence	No	Ref	Ref	Ref
	Yes	1.22 (1.19–1.25)	1.23 (1.18–1.28)	1.37 (1.25–1.49)
Tobacco Use	No	Ref	Ref	Ref
	Yes	1.09 (1.08–1.11)	1.12 (1.09–1.14)	1.17 (1.11–1.23)
Rurality	Most Urban	Ref	Ref	Ref
	Urban	1.04 (1.03–1.06)	1.05 (1.04–1.07)	1.06 (1.02–1.10)
	Rural	1.01 (1.00–1.02)	1.07 (1.05–1.08)	1.13 (1.08–1.18)
	Most Rural	1.01 (0.97–1.05)	1.12 (1.10–1.15)	1.36 (1.27–1.45)
Age at first ESRD service date	18–39	Ref	Ref	Ref
	40–65	2.05 (2.02–2.09)	2.00 (1.95–2.06)	2.16 (2.03–2.30)
	Over 65	4.29 (4.22–4.37)	4.13 (4.01–4.25)	4.43 (4.16–4.71)

^a^HR (95% CI) = Hazard ratio (95% Confidence Interval).

When we ran overall models stratified by age, survival differed by age (not shown). When we further stratified the hospital distance-stratified model by age, we found the same negative association between better EQIs and survival for patients under 10 miles from a hospital (S1 Fig, S1 Table). The strongest association was for the under 40 age group where median survival in the best EQI percentile was 6.30 years and increases to 13.21 years in the worst EQI percentile. As we found in the overall hospital distance-stratified model, for patients over 20 miles from a hospital there is a positive association between survival time and better EQIs (S1 Fig). This association is greatest in the 40–65 age group with a median and 90^th^ percentile survival of 5.53 and over 14 years (survival beyond 2000–2013 study time) in the best EQI percentile and 4.46 and 12.73 years in the worst EQI percentile.

In overall models stratified by rurality, we did not find differing survival (not shown). Because both the environment and distance to the nearest hospital could be influenced by rurality, we stratified the hospital distance-stratified model by rurality groups. The same general pattern that we found for the overall hospital distance-stratified model and age and hospital distance-stratified model held across hospital distance and rural group-stratified models (S2 Fig, S2 Table).

To determine if any of the individual EQI domains were contributing to the pattern we found for the total EQI hospital distance-stratified model, we stratified each domain of the EQI by the distance to the nearest hospital. While there was no substantial difference between the different hospital groups for the air, water, and land EQI domains (not shown), the built and sociodemographic EQI domains had a pattern similar to the total EQI hospital distance-stratified model (Table 4). For the sociodemographic domain, patients within 10 miles of the nearest hospital had increased survival in the worst EQI percentile compared to the best percentile (HR = 0.96, 95% CI = 0.93–0.98) and patients over 20 miles from the nearest hospital had decreased survival in the worst EQI percentile compared to the best percentile (HR = 1.17, 95% CI = 1.09–1.27). This same pattern also occurred in the built domain, but the results were not significant.

**Table 4:** Hazard ratios for ESRD and built and sociodemographic EQI domain stratified by distance to the nearest hospital (1,092,281 patients, 2000–2013).

		**Built EQI**		
		**Under 10 miles**	**10–20 miles**	**Over 20 miles**
		HR (95% CI)^a^	HR (95% CI)	HR (95% CI)
EQI Category	EQI 0–5% (best)	Ref	Ref	Ref
	EQI 5–20%	0.98 (0.87–1.1)	1 (0.95–1.05)	1.08 (0.97–1.21)
	EQI 20–40%	0.93 (0.83–1.04)	0.96 (0.91–1.01)	0.97 (0.88–1.07)
	EQI 40–60%	0.93 (0.83–1.05)	0.88 (0.84–0.93)	0.97 (0.87–1.07)
	EQI 60–80%	0.94 (0.84–1.05)	0.96 (0.91–1.01)	1.08 (0.98–1.19)
	EQI 80–95%	0.88 (0.78–0.99)	0.92 (0.87–0.97)	1.08 (0.97–1.2)
	EQI 95–100% (worst)	0.92 (0.82–1.04)	0.91 (0.86–0.96)	1.07 (0.96–1.19)
		**Sociodemographic EQI**		
		**Under 10 miles**	**10–20 miles**	**Over 20 miles**
		HR (95% CI)	HR (95% CI)	HR (95% CI)
EQI Category	EQI 0–5% (best)	Ref	Ref	Ref
	EQI 5–20%	1.05 (1.02–1.08)	1.12 (1.09–1.15)	1.11 (1.06–1.17)
	EQI 20–40%	0.93 (0.9–0.95)	1.15 (1.12–1.18)	1.18 (1.12–1.24)
	EQI 40–60%	1.04 (1.01–1.06)	1.15 (1.12–1.18)	1.15 (1.1–1.2)
	EQI 60–80%	1.02 (0.99–1.05)	1.14 (1.11–1.17)	1.22 (1.17–1.27)
	EQI 80–95%	0.99 (0.96–1.02)	1.14 (1.11–1.17)	1.29 (1.2–1.39)
	EQI 95–100% (worst)	0.96 (0.93–0.98)	1.12 (1.07–1.17)	1.17 (1.09–1.27)

^a^HR (95% CI) = Hazard ratio (95% Confidence Interval.

## Discussion

We found that survival time for patients with ESRD was higher among those residing farther from the nearest hospital. This association held across different rural and urban continua. Compared to living within ten miles of the nearest hospital, patients living over 20 miles away had a median increased survival of 3.4 months. While this observation was unexpected given the importance of continuous access to care for patients with ESRD, other studies have also found that living closer to a hospital does not always improve survival [21,27,65] and may lead to overutilization [19,66]. One possibility for this discrepancy is that people living farther from a hospital are more likely to utilize primary and specialist care facilities more regularly which can improve overall health [67,68]. Because we did not include any variables accounting for care or insurance prior to ESRD development, we could be missing an important contributing factor related to hospital utilization [19,65]. Future efforts to elucidate the relationship between access to care and survival time with ESRD should incorporate an additional metric for this important sociodemographic contribution to survival.

When we analyzed the effect that environmental quality has on survival time for patients with ESRD using the EQI, we did not find any clear association across EQI strata. However, when the full model was adjusted for EQI and stratified by distance to the nearest hospital, we found an association between survival time and the EQI. Patients living within 10 miles of the nearest hospital had a 6 month increase in median survival time when living in the worst EQI percentile as compared to the best EQI percentile. This association flipped for patients living over 20 miles from the nearest hospital with a 9 month increase in median survival time when living in the best EQI percentile as compared to the worst EQI percentile. This pattern also holds across different age groups and different rural and urban categories. Interestingly, this pattern also held for the built and sociodemographic domains of the EQI, suggesting that these two domains may be contributing to the effect that we observe. One possibility for this unexpected pattern is that the EQI inadequately captures these components of the environment that have the most influence on ESRD survival. Some domains of the EQI are better represented than others, such as the air domain (87 variables) and the water domain (80 variables). However the built domain (14 variables) and sociodemographic domain (12 variables) have less data owing to fewer sources being available for use [40]. Based on our findings with access to care, the built domain may be an important influence in survival for patients with ESRD. Further, because poorer socioeconomic status is a risk factor for ESRD [2,28,29], the sociodemographic domain is likely to be important in capturing the environmental influence on ESRD. The sociodemographic domain has variables related to income and education [41], both of which have been highlighted as important social factors in kidney disease [5,29]. However, the generalized measures at the county level of the EQI may not be of sufficient resolution to capture social gradients that likely influence survival in patients with ESRD. Additionally, other cultural and behavioral factors at the individual level such as familial support and attitudes towards health [5,8,69] are not captured in this county-level measure of socioeconomic status.

Another possible contributor to the unclear association between hospital distance, the EQI, and ESRD survival time is that the environment may not be resolved well at the county level. The EQI was constructed at the county level as this was the geographic level at which most data was consistently available [40]. However, county size is varied across the US with the 3,111 counties in our study ranging from 6.3 mi^2^ in Fairfax City, Virginia to 146,000 mi^2^ in Yukon-Koyukuk, Alaska. Larger counties also have an apparent association with better EQIs. Less than 1% of the counties in our study are over 10,000 mi^2^, yet 70% of them are located in the top three EQI percentiles. Further, because individual level behaviors are crucial in determining neighborhood health, refining the geographic region below the county level may be more important for diseases where the sociodemographic domain plays a stronger role [69,70]. While a preliminary comparison of the full model stratified by distance to the nearest hospital and county size did not change the pattern we found with all counties in one model, refining the level of analysis to be at a geographic scale finer than the county level may better elucidate elements of the environment influencing survival.

An advantage of our study is our determination of access to healthcare for a geographic region. While the EQI does have a variable in the built domain to describe healthcare, the metric is calculated differently than ours and only counties with healthcare-related businesses include this measure [71]. By determining distance to the nearest hospital regardless of county lines, we are providing an improved measure of access to care for each county than what is already contained within the EQI. Further, by determining the distance at the county level rather than directly from a patient’s address, we are enabling distances to be calculated relative to a number of different locations within a person’s county (e.g., medical visits may be made from a place of work rather than a place of residence [47]). Another advantage of our study is that it focuses only on individuals who remained in one county for the duration of their lifetime. Focusing our study in this way eliminated almost a quarter of the patients we could have included in our study, however despite small differences in survival time the demographic makeup of these patients was comparable to those of the patients that remained. Because early detection of ESRD is expected to delay adverse outcomes [15–17], survival time for ESRD would depend on the environment and access to care before diagnosis. While an individual county’s average hospital distance and EQI would be unlikely to remain constant over the course of an individual’s lifetime, narrowing the study in this manner ensures that all patients who remained within one county experienced a similar environmental change.

One key assumption of our study is that the average distance to the nearest hospital is an equally valid metric in both urban and rural regions of the US. While numerous studies have found the Euclidian distance to be highly correlated with road network distances when assessing access to healthcare [47–53], exception can be made in more rural areas as the geographical barriers to healthcare may be underestimated [48,52]. Despite this, we still found the same associations in both urban and rural counties. Additionally, the hospital data we used were registered with Medicare, so it is possible that we are missing some other hospitals that would have been utilized by the individuals in our study.

Another limitation of our study is the variation between the timeframes of data we are using. The patient data from USRDS covers the years 2000–2013 while the EQI data covers the years 2000–2005. However, several sources of the data included in the EQI are derived from census data which are updated every ten years, meaning that multiple elements of the EQI would remain the same at least through 2010 [40]. We used a 2018 release of hospital data from the Medicare website [56] which regularly updates hospital information and contains archived hospital data back to 2005. However, these archived data comprise several addresses that no longer exist making it difficult to pinpoint even approximate locations for many hospitals. Despite our hospital dataset being outside the timeframe under study, we find it representative as hospital closures and openings occurred within the 2000–2013 timeframe. Comparing hospital provider IDs between 2005 and 2013, we found 10% of the hospitals from 2005 missing from the 2013 data and 24% of the hospitals from 2013 data missing from the 2005 data. Comparing the 2013 data to our 2018 hospital data, 8% of the respective hospitals were missing from the other dataset. Future studies could analyze whether these hospital closures and openings had any influence on patient survival. Additionally, given the 14 year timeframe of our study, changes in the treatment of ESRD during that time as well as implementation of the Affordable Care Act could be important factors to consider when trying to better elucidate the associations between patient survival and access to healthcare [72].

## Conclusions

Both access to care and the broader environment were important factors to consider in survival for patients with ESRD. The influence of the environment on survival for patients with ESRD has not been explored in depth before and the additional level of influence related to access to care emphasizes the importance of considering multiple factors when looking at survival for diseases with such poor prognoses. Our study found an unexpected association among hospital distance, the overall environment, and ESRD survival time, and elucidated that the built and sociodemographic environmental domains may be contributing to this association. Future efforts should explore additional factors related to socioeconomic standing and analyze influences of the environment at geographic scales smaller than the county level. Other factors related to access to care such as insurance status prior to diagnosis and use of primary versus secondary care should also be explored for their influences on ESRD progression.

## Supporting information

S1 Fig Adjusted survival curves for all patients developed using the full model, stratified by distance to the nearest hospital and age at first ESRD service date. Quantiles were generated using the total EQI. (Left) Patients with nearest hospital under 10 miles away, (Right) Patients with nearest hospital over 20 miles away. (Top) Patients aged 18–39, (Middle) Patients aged 40–65, (Bottom) Patients over 65.
(EPS)

S2 Fig Adjusted survival curves for all patients developed using the full model, stratified by distance to the nearest hospital and rurality. Quantiles were generated using the total EQI. (Left) Patients with nearest hospital under 10 miles away, (Right) Patients with nearest hospital over 20 miles away. (Top) Urban counties, (Bottom) Rural counties.
(EPS)

S1 Table Hazard ratios for ESRD and total EQI stratified by age and distance to the nearest hospital (1,092,281 patients, 2000–2013). ^a^HR (95% CI) = Hazard ratio (95% Confidence Interval).
(DOCX)

S2 Table Hazard ratios for ESRD and total EQI stratified by rural versus urban location and distance to the nearest hospital (1,092,281 patients, 2000–2013). ^a^HR (95% CI) = Hazard ratio (95% Confidence Interval).
(DOCX)
