# Supplementary material for: Associations between access to healthcare, environmental quality, and end-stage renal disease survival time: Proportional-hazards models of over 1,000,000 people over 14 years
Source: PLoS One. 2019 Mar 21;14(3):e0214094. doi: 10.1371/journal.pone.0214094 (PMC6428249; doi:10.1371/journal.pone.0214094)
Supplement: S1 Table — aHR (95% CI) = Hazard ratio (95% Confidence Interval). (DOCX) [file pone.0214094.s003.docx]

|  |  | **Age 18-39** | |
| --- | --- | --- | --- |
|  |  | **Under 10 miles** | **Over 20 miles** |
|  |  | HR (95% CI)^a^ | HR (95% CI) |
| EQI Category | EQI 0-5% (best) | Ref | Ref |
|  | EQI 5-20% | 0.73 (0.46-1.14) | 1.22 (0.79-1.90) |
|  | EQI 20-40% | 0.74 (0.47-1.16) | 1.07 (0.70-1.62) |
|  | EQI 40-60% | 0.72 (0.46-1.13) | 1.02 (0.66-1.58) |
|  | EQI 60-80% | 0.70 (0.45-1.09) | 1.24 (0.80-1.91) |
|  | EQI 80-95% | 0.65 (0.41-1.01) | 1.01 (0.65-1.55) |
|  | EQI 95-100% (worst) | 0.52 (0.33-0.81) | 1.11 (0.64-1.93) |
|  |  | **Age 40-65** | |
|  |  | **Under 10 miles** | **Over 20 miles** |
|  |  | HR (95% CI) | HR (95% CI) |
| EQI Category | EQI 0-5% (best) | Ref | Ref |
|  | EQI 5-20% | 1.11 (0.94-1.31) | 1.14 (1.00-1.31) |
|  | EQI 20-40% | 1.10 (0.93-1.29) | 1.07 (0.95-1.21) |
|  | EQI 40-60% | 1.11 (0.94-1.30) | 1.14 (1.01-1.30) |
|  | EQI 60-80% | 1.06 (0.90-1.25) | 1.17 (1.03-1.33) |
|  | EQI 80-95% | 1.04 (0.89-1.23) | 1.16 (1.02-1.32) |
|  | EQI 95-100% (worst) | 0.92 (0.78-1.09) | 1.27 (1.08-1.50) |
|  |  | **Age Over 65** | |
|  |  | **Under 10 miles** | **Over 20 miles** |
|  |  | HR (95% CI) | HR (95% CI) |
| EQI Category | EQI 0-5% (best) | Ref | Ref |
|  | EQI 5-20% | 0.97 (0.85-1.12) | 1.11 (0.98-1.25) |
|  | EQI 20-40% | 0.93 (0.81-1.06) | 1.05 (0.94-1.18) |
|  | EQI 40-60% | 0.93 (0.81-1.06) | 1.21 (1.08-1.36) |
|  | EQI 60-80% | 0.90 (0.79-1.03) | 1.13 (1.01-1.27) |
|  | EQI 80-95% | 0.89 (0.78-1.02) | 1.19 (1.06-1.33) |
|  | EQI 95-100% (worst) | 0.84 (0.73-0.96) | 1.21 (1.06-1.39) |
