# Supplementary material for: Associations between access to healthcare, environmental quality, and end-stage renal disease survival time: Proportional-hazards models of over 1,000,000 people over 14 years
Source: PLoS One. 2019 Mar 21;14(3):e0214094. doi: 10.1371/journal.pone.0214094 (PMC6428249; doi:10.1371/journal.pone.0214094)
Supplement: S2 Table — aHR (95% CI) = Hazard ratio (95% Confidence Interval). (DOCX) [file pone.0214094.s004.docx]

|  |  | **Urban** | |
| --- | --- | --- | --- |
|  |  | **Under 10 miles** | **Over 20 miles** |
|  |  | HR (95% CI)^a^ | HR (95% CI) |
| EQI Category | EQI 0-5% (best) | Ref | Ref |
|  | EQI 5-20% | 0.93 (0.88-0.98) | 0.99 (0.89-1.09) |
|  | EQI 20-40% | 0.93 (0.89-0.98) | 0.98 (0.89-1.08) |
|  | EQI 40-60% | 0.93 (0.88-0.98) | 1.02 (0.93-1.12) |
|  | EQI 60-80% | 0.87 (0.83-0.92) | 1.01 (0.92-1.11) |
|  | EQI 80-95% | 0.87 (0.83-0.92) | 1.03 (0.94-1.13) |
|  | EQI 95-100% (worst) | 0.79 (0.75-0.83) | 1.09 (0.97-1.23) |
|  |  | **Rural** | |
|  |  | **Under 10 miles** | **Over 20 miles** |
|  |  | HR (95% CI) | HR (95% CI) |
| EQI Category | EQI 0-5% (best) | Ref | Ref |
|  | EQI 5-20% | 1.01 (0.94-1.08) | 1.14 (1.01-1.28) |
|  | EQI 20-40% | 0.99 (0.92-1.06) | 1.17 (1.06-1.29) |
|  | EQI 40-60% | 0.99 (0.92-1.06) | 1.20 (1.08-1.34) |
|  | EQI 60-80% | 0.96 (0.89-1.02) | 1.08 (0.97-1.20) |
|  | EQI 80-95% | 0.92 (0.86-0.99) | 1.20 (1.05-1.37) |
|  | EQI 95-100% (worst) | 0.92 (0.85-0.99) | 1.21 (0.96-1.53) |
